# Polyglutamic Acid as an Antiviral Agent: Mechanistic and Structural Insights

**DOI:** 10.3390/pharmaceutics17101296

**Published:** 2025-10-02

**Authors:** Ya-Na Wu, Shang-Rung Wu

**Affiliations:** 1 School of Dentistry & Institute of Oral Medicine, College of Medicine, National Cheng Kung University, Tainan City 701, Taiwan; z9508182@ncku.edu.tw; 2 Institute of Basic Medical Sciences, College of Medicine, National Cheng Kung University, Tainan City 701, Taiwan

**Keywords:** antiviral, γ-poly(glutamic acid), polyglutamate, polyglutamic acid, PGA, structural biology, CD14

## Abstract

Poly-γ-glutamic acid (γ-PGA), also known as polyglutamate, is a naturally derived polymer produced by *Bacillus* species that has demonstrated antiviral properties. Growing evidence from preclinical and clinical studies supports its therapeutic potential against various viral infections, highlighting both effective antiviral activity and a favorable safety profile. This review emphasizes current findings on the antiviral mechanisms of γ-PGA, including its ability to interfere with viral entry and to activate serial immune signaling pathways, with additional insights from structural biology. Collectively, γ-PGA represents a promising biomaterial for the development of future broad-spectrum antiviral strategies and applications.

## 1. Introduction

Current viral outbreaks underscore the critical need for broad-spectrum antiviral agents that can tackle multiple viruses. Traditional therapies are often designed for specific viral infections, thus limiting their applications. In contrast, broad-spectrum antivirals ideally provide protection against diverse strains and can be deployed rapidly during outbreaks. Among the various biomaterials explored for their antiviral potential, including glycosaminoglycans, algal polysaccharides, mucin, and chitosan [1]. Poly-γ-glutamic acid (γ-PGA) has recently emerged as one of the most promising candidates and has been reviewed in detail [2,3].

**Scheme 1:**
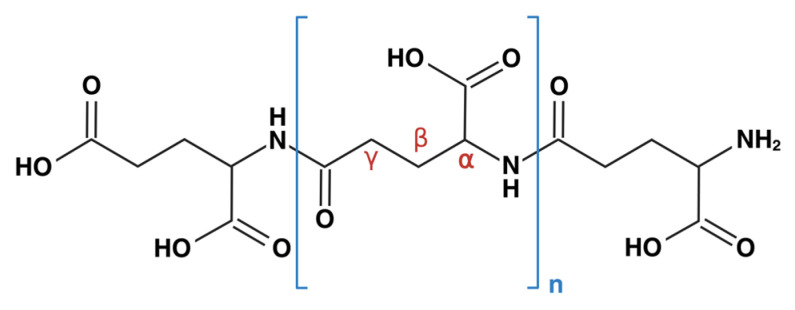
Chemical Structure of γ-PGA. γ-PGA is an extracellular polypeptide composed of glutamic acid units connected by γ-amide linkages. It is naturally produced by various *Bacillus* species.

γ-PGA is an extracellular polypeptide produced by several *Bacillus* species and is composed of glutamic acid units linked by γ-amide bonds (Scheme 1). It is reported as a polyanionic, biodegradable, low-immunogenic [4], and edible polymer [5]. Advances in biotechnology have allowed precise manipulation of γ-PGA’s molecular weight, ranging from several kilodaltons (kDa) up to over 8000 kDa [1,6]. γ-PGA, widely used in food and cosmetic products, has demonstrated a strong safety profile to date [5,7]. Beyond its broad commercial use, γ-PGA offers advantageous properties such as hydrophilicity, biocompatibility, and biodegradability. γ-PGA also enables the formation of nanoparticles or complexes with therapeutic agents via ionic interactions or self-assembly, thereby facilitating controlled release [8]. Its ability to modulate immune responses, activate multiple immune cell populations, and induce cytokine production further highlights its potential in antitumor and antiviral therapies [9,10,11].

Although the antiviral activity of γ-PGA was first reported more than a decade ago, several recent reviews have primarily focused on its production [12,13,14], biomedical applications [15], commercial uses [12], and its roles in vaccine platforms, both as a nanoparticle carrier for antigen delivery and as an adjuvant [16]. These studies reflect γ-PGA’s broad potential and versatility. In contrast, this review specifically examines the intrinsic antiviral properties of purified γ-PGA. To clearly assess its mechanisms, we include only studies employing non-engineered γ-PGA, excluding those involving chemically modified derivatives, vaccine-adjuvant formulations, or antigen delivery platforms. Here, we synthesize current knowledge on the antiviral mechanisms of γ-PGA, immune signaling pathways it engages, and structural determinants of its activity, while also discussing its prospects as a broad-spectrum and versatile antiviral agent.

## 2. Antiviral Effects of γ-PGA

**Figure 1:**
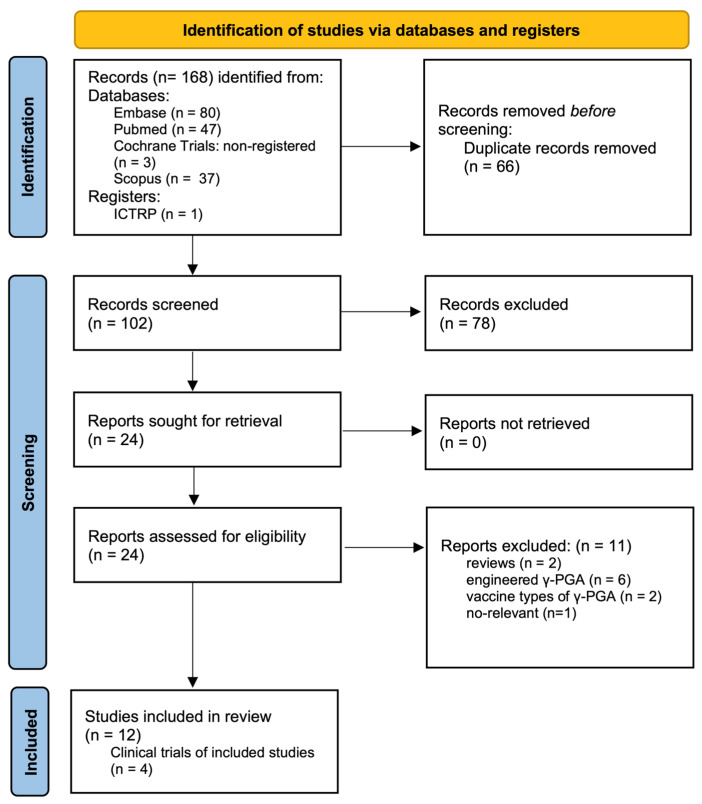
PRISMA 2020 flow diagram depicting selection of studies.

Figure 1 presents a flow diagram outlining the selection procedure for this review. We identified 12 eligible studies that employed non-engineered γ-PGA to evaluate its antiviral efficacy and underlying mechanisms. The antiviral activity of γ-PGA has been extensively investigated and demonstrated to be effective both in vitro and in vivo against a wide range of viruses (Table 1), including herpes simplex virus type 1 (HSV-1) [17,18], herpes simplex virus type 2 (HSV-2) [17,19,20], Pseudorabies virus (PRV) [17], vesicular stomatitis virus (VSV) [17], Murine norovirus (MNV) [21], influenza A virus (specifically the H1N1 [22,23] and H5N2 [22] subtypes), porcine reproductive and respiratory syndrome virus (PRRSV) [23], Newcastle disease virus (NDV) [24], hepatitis C virus (HCV) [25], and severe acute respiratory syndrome coronavirus (SARS-CoV) [25].

Three major antiviral mechanisms have been proposed for γ-PGA, each of which will be discussed in the following sections: (1) interference with viral entry into host cells, (2) prophylactic priming of innate immune responses before infection, and (3) therapeutic activation of antiviral immunity after viral exposure. Together, these mechanisms highlight γ-PGA’s potential not only as a preventive agent but also as a viable therapeutic option post-infection, underscoring its clinical relevance in the management of viral diseases.

### 2.1. Interference in Viral Entry with γ-PGA Containing Exopolymers

Sánchez-León et al. demonstrated that γ-PGA containing exopolymers, when co-incubated with HSV-1, HSV-2, VSV, and PRV for an hour before infection, reduced viral titers by approximately five orders of magnitude compared with the mock-treated control group [17]. Similar inhibitory effects against HSV-2 were reported by Polie et al. [19]. However, γ-PGA showed no effect on non-enveloped viruses such as Minute virus of mice, suggesting a mechanism specific to membrane-dependent viral entry [17]. As the tested preparation contained both γ-PGA and teichoic acid, the exact contribution of γ-PGA alone remains to be clarified. Moreover, studies of viral entry inhibition by γ-PGA–containing exopolymers have been limited to in vitro settings, with no supporting in vivo data. Nevertheless, these findings highlight the potential of γ-PGA as a preventive tool to reduce infectious risk prior to viral exposure.

**Scheme 2:**
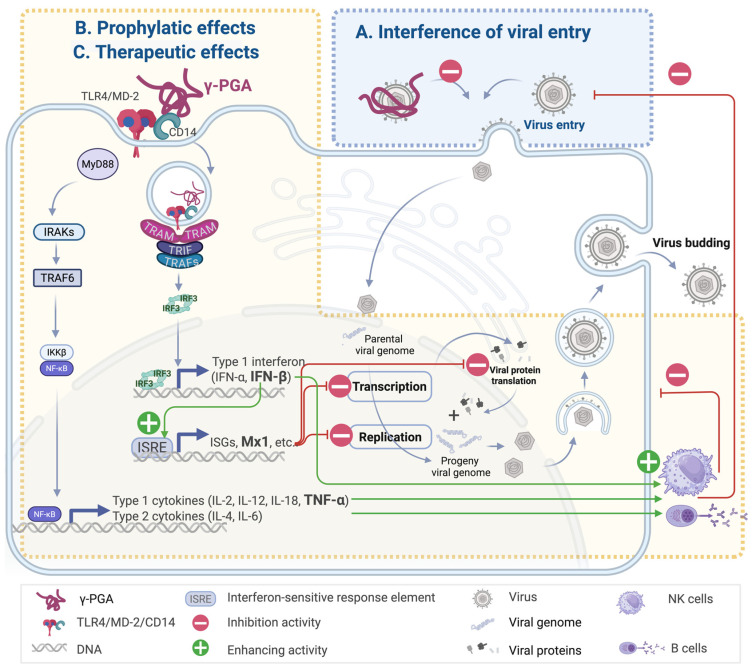
Multifaceted Antiviral Mechanisms of γ-PGA. Γ-PGA exerts antiviral effects through three key mechanisms. (**A**) Interference of viral entry: γ-PGA-containing exopolymers block adsorption of enveloped viruses to host cells in a dose-dependent manner. (**B**) Prophylactic immune priming: Pretreatment with γ-PGA binds CD14 and activates TLR4-mediated IFN-β signaling, priming cells for a rapid response against impending viral infections. (**C**) Therapeutic immune activation: Post-exposure administration of γ-PGA upregulates of type 1 cytokines (e.g., TNF-α, IL-12), enhancing cell-mediated immunity through activation of NK cells and cytotoxic T lymphocytes. At later stages it also promotes stronger type 2 cytokine responses, supporting humoral immunity. Collectively, these effects improve survival rates in animal models. (Abbreviations: γ-PGA, polyglutamic acid; IFN-α, interferon-α; IFN-β, interferon-β; IKKβ, IκB kinase β; IL-12, interleukin-12; IL-18, interleukin-18; IL-1β, interleukin-1β; IL-2, interleukin-2; IL-4, interleukin-4; IL-6, interleukin-6; IRF3, interferon regulatory factor 3; IRAKs, IL-1 receptor kinases; ISGs, interferon-stimulated genes; ISRE, interferon-stimulated response element; MD-2, myeloid differentiation factor 2; Mx1, myxovirus resistance 1; NF-κB, nuclear factor κB; NK cells, natural killer cells; TLR4, Toll-like receptor 4; TNF-α, tumor necrosis factor-α; TRAF6, tumor necrosis factor receptor factor 6; TRAFs, tumor necrosis factor receptor factors; TRIF, TIR adaptor inducing IFN-β; TRAM, TRIF-related adaptor).

Taken together, γ-PGA-containing exopolymers demonstrate significant antiviral potential by interfering with viral adsorption onto host cells in a dose-dependent manner (Scheme 2), as shown in limited in vitro studies. Further research is needed to (1) isolate the specific role of γ-PGA from exopolymers, (2) delineate the molecular basis of this entry-blocking effect, and (3) evaluate its potential as a preventive antiviral strategy in vivo.

### 2.2. Prophylactic Effect of γ-PGA

Several studies have reported that pretreatment with γ-PGA provides strong protection both in vitro and in vivo. Cells pre-exposed to γ-PGA for 2 [18], 6 [21], or 12 h [22,24,25] showed more than 70% reduction in viral replication. These priming treatments reduced viral replication to 30% or less for SARS-CoV [25], HCV [25], NDV [24], HSV-1 [18] and MNV [21]. Remarkably, even a short 2 h pretreatment reduced viral yield to approximately 20% at 48 h post-inoculation. In animal models, γ-PGA pretreatment led to nearly a 1000-fold reduction in influenza A virus titers in the lungs [22] and significantly decreased MNV titers in mouse ileum [21].

At the molecular level, γ-PGA binds cluster of differentiation 14 (CD14) and triggers toll-like receptor 4 (TLR4)-mediated interferon beta (IFN-β) response [25] (Scheme 2). Following γ-PGA treatment, cells exhibited rapid upregulation of nuclear translocation of interferon regulatory factor 3 (IRF3) [24], accompanied by increased expression of IFN-β [18,21,22,24] and myxovirus resistance protein 1 [22,24], both critical molecules involved in innate immune activation and antiviral defense. Subsequently, additional antiviral mediators such as interferon-stimulated genes (ISGs), including ubiquitin-like antiviral proteins, increase [24]. Some studies note a lipopolysaccharide (LPS)-like cytokine profile [18,24], whereas Lee et al. found selective IFN-β induction without detectable increases in proinflammatory cytokines in blood samples of mice administered γ-PGA orally [21]. This discrepancy may stem from differing time points, protocols, or cell types used across studies, necessitating further investigation.

In vivo, daily oral administration of γ-PGA once for five days significantly increased serum IFN-β levels in mice by day 3 [21]. This prophylactic effect is attributed to the priming of IFN-β and downstream antiviral mediators, which condition immune cells for a rapid response prior to viral challenge. In a study by Poli et al. [19], co-treatment of peripheral blood mononuclear cells (PBMCs) or WISH cells with γ-PGA and HSV-2 reduced viral count to approximately 25% within 24 h. The active prophylactic γ-PGA used in these studies ranged from 890 to 1000 kDa [18], 2000 kDa [21], to 3000 kDa [22,24]. When the molecular weight was too low (~500 kDa) or too high (~5000 kDa), γ-PGA failed to induce sufficient IFN-β expression, leading to diminished antiviral efficacy [21,25].

### 2.3. Therapeutic Potential of γ-PGA Following Viral Exposure

Beyond entry prevention and its prophylaxis, γ-PGA exhibits therapeutic antiviral activity even after viral exposure. Notable effects appear against Influenza A [26], MNV [21], SARS-CoV [25], and PRRSV [23] in vitro and in vivo models. For example, influenza-infected mice exhibited approximately 50% survival by day 8 to day 9, whereas intranasal administration of γ-PGA resulted in 100% survival by day 14, even across two distinct influenza A strains [26]. Similarly, intramuscular administration of γ-PGA in pigs on day 0, 3, or 7 post-PRRSV challenge reduced viremia and lung lesion severity [23].

In therapeutic settings, Kim et al. [26] and Seo et al. [23] reported notable upregulation of IFN-β along with several responsive cytokines, including tumor necrosis factor-alpha (TNF-α), interferon alpha (IFN-α), interferon gamma (IFN-γ), interleukin-2 (IL-2), interleukin-4 (IL-4), interleukin-6 (IL-6), and interleukin-12 (IL-12), as shown in Scheme 2. Kim et al. [26] further demonstrated enhanced activation of natural killer (NK) cells and influenza antigen-specific cytotoxic T lymphocytes in vivo. These findings position γ-PGA as a promising therapeutic agent capable of bolstering antiviral immunity post-exposure, highlighting its clinical applicability.

## 3. Clinical Trials

Several clinical trials have demonstrated the therapeutic potential of γ-PGA in diverse health conditions, including delayed gastric emptying to improve glycemic control [27,28], reduced cholesterol and fat accumulation [29], and enhanced calcium absorption for bone health [30]. A multicenter, randomized, double-blind, phase II trial evaluated the antiviral and antitumor effects of γ-PGA in women with cervical intraepithelial neoplasia 1 [31]. In this study, 195 participants received either 1500 mg oral γ-PGA or placebo daily for four weeks, with a follow-up period of 12 weeks. Although no significant changes in activity of NK cells were observed, the γ-PGA group exhibited a significantly higher rate of histologic remission (42.4% vs. 27.1%, *p* = 0.018) and human papillomavirus clearance (43.5% vs. 26.7%, *p* = 0.026) compared to placebo. NK cells activity increased at weeks 4 and 8, though not to a statistically significant degree.

The trial supports γ-PGA as a promising, non-invasive oral therapeutic agent for cervical intraepithelial neoplasia 1 and high-risk human papillomavirus infections, with a favorable safety profile in the short term. While γ-PGA has been reported to induce proinflammatory cytokines in some reports [18,19,21,23], it consistently shows good tolerability, with only mild placebo-like side effects and no serious adverse events even at high oral doses within a 12-week period [31,32]. Nevertheless, further clinical trials and post-market surveillance are required to address key limitations: (1) the sample size was not sufficient to represent the general population, (2) the follow-up period was too short to assess long-term outcomes, and (3) the study design did not account for variability in antiviral efficacy across genders, races, and genetic backgrounds.

Previous studies have also explored the antiviral properties of γ-PGA, with a particular focus on HSV-1 [17,18], HSV-2 [17,19], and influenza virus [22,26] infections in various in vitro models, as summarized in Table 1. Among these, the protective role of γ-PGA against influenza has been investigated in macrophage-lineage cells and mouse models, suggesting influenza as a promising target for future preclinical and clinical trials. For HSV-1 and HSV-2, antiviral effects have been reported across several cell types, including white blood cells, cervical epithelial cells, and other epithelial cell lines. Taken together, influenza virus, HSV-1, and HSV-2 represent strong candidates for clinical research aimed at validating the antiviral efficacy of γ-PGA, which could pave the way for its development as a broad-spectrum therapeutic supported by robust clinical evidence.

## 4. Pharmaceutical Considerations for γ-PGA

### 4.1. Stability and Bioavailability

γ-PGA is highly resistant to hydrolysis, showing minimal degradation at room temperature in the absence of enzymes [33]. Even in vivo, human gastric enzymes do not target the γ-peptide bond. Instead, a fraction of γ-PGA is degraded by gut microbial enzymes into absorbable molecules, while the intact polymer does not appear to cross into systemic circulation [34]. When administered orally or intranasally, high–molecular weight γ-PGA largely remains in the gastrointestinal or nasal mucosa, where it enhances nutrient absorption and stimulates gut-associated immune tissues. Intranasal formulations are typically prepared as solutions or mucoadhesive blends, adhering to the nasal mucosa and potently stimulating local innate immunity in animal models [26]. Oral delivery formats include syrups [31], powders [35], and hydrogels [36].

### 4.2. Safety and Regulations

Unmodified γ-PGA is generally considered safe and biocompatible, as demonstrated in numerous in vitro and in vivo studies [25] and phase I/II clinical trials conducted over the past decade [31,32]. γ-PGA has been extensively evaluated via oral, intranasal, subcutaneous, and intravenous routes, and has consistently been shown to be safe and non-immunogenic, reviewed by Elbanna et al. [12]. Regulatory agencies have also recognized its safety: the U.S. Food and Drug Administration lists γ-PGA as Generally Recognized as Safe (GRN No. 339) for use in foods, and the Ministry of Food and Drug Safety in South Korea has approved γ-PGA as an individual health functional food ingredient for immune function (No. 200800200116). Regulators in multiple countries have vetted γ-PGA for use in foods, dietary supplements, and clinical trials, with no major safety concerns reported.

### 4.3. General Pharmackinetics

Following intravenous administration, γ-PGA exhibits a short plasma half-life ranging from minutes to a few hours in animal studies, depending on dose and molecular size [37]. Sutherland et al. [37] reported that its volume of distribution approximates plasma volume or is slightly higher. After distribution, γ-PGA undergoes biphasic clearance in the liver and spleen, consisting of a rapid phase followed by a slower phase. In mice, the maximal clearance was about 140 µg/h for a 20 g mouse. High doses in mice yielded hepatic half-lives of 6 h in the fast phase and 4–8 days in the slow phase. A significant proportion of the polymer persisted in the liver for 1–2 weeks post-injection but was nearly eliminated by 3 weeks.

## 5. Downstream Molecular Signaling Pathways of γ-PGA

Innate immune receptors detect pathogen-associated molecular patterns via pattern-recognition receptors [38]. Among the pattern-recognition receptors, Toll-like receptors are key in host defense, e.g., Toll-like receptor 2 detects lipopeptides, Toll-like receptors 3 identifies viral double-stranded RNA, and TLR4 senses LPS. γ-PGA exerts antiviral effects by triggering TLR4-dependent immune responses (Scheme 2) [9]. γ-PGA binds CD14 to engage the TLR4-myeloid differentiation factor 2 (MD-2) complex, which activates two downstream cascades: (1) the myeloid differentiation factor 88 (MyD88)-dependent pathway and (2) the Toll/IL-1 receptor-domain-containing adaptor-inducing IFN-β (TRIF)-dependent pathway [24,25].

In the MyD88-dependent pathway, the TLR4-MyD88 complex recruits IL-1 receptor-associated kinases, particularly IL-1 receptor-associated kinases 1 and 4. The activation of these kinases leads to recruitment of tumor necrosis factor receptor-associated factor 6. Ultimately, this signaling induces proinflammatory cytokines such as IL-1β [18,24], type 1 cytokines including IL-2 [23], IL-12 [19,26], IL-18 [19], and TNF-α [18,19,22,23,26], as well as type 2 cytokines such as IL-4 [23] and IL-6 [18,23,24]. This rapid cytokine burst aids antiviral defense [39].

The TRIF-dependent pathway promotes IRF3 dimerization and nuclear translocation, which drives IFN-β expression [18,21,22,23,24,25,26] and induces interferon-stimulated genes (ISGs) [22,24] such as interferon regulatory factor 7 [24], myxovirus resistance protein 1 [22,24], guanylate-binding proteins [22,24], ISG-56 [22], and 2′-5′-oligoadenylate synthetases [22,25]. This pathway is essential for establishing antiviral states and for dendritic cell maturation. Type I interferons (including IFN-α and IFN-β) elicited through this pathway play a central role in mounting antiviral responses [25,40]. myxovirus resistance protein 1, for instance, is critical in inhibiting replication across various RNA and DNA viruses [41]. “-”-oligoadenylate synthetase confers protection against diverse RNA and DNA viral pathogens [42], and ISG-56 significantly contributes to restricting viral replication [43].

An in vivo study conducted by Seo et al. [23] showed that a five-day oral γ-PGA treatment led to increased mRNA expression of type I interferons (IFN-α and IFN-β), type II interferons (IFN-γ), TNF-α, type 1 cytokines (IL-2), and type 2 cytokines (IL-4) in PBMCs. This response induced an antiviral state and enhanced the elimination of infected cells. Notably, γ-PGA rapidly induced expression of type I interferons and type 1 cytokines from day 0, followed by type 2 cytokines by day 3 post-treatment. These findings align with earlier reports [44], suggesting that γ-PGA triggers both innate and adaptive immune responses.

γ-PGA thereby activates both type I and type II interferon responses: IFN-β production likely arises from direct TLR4-IRF3 signaling in specific cell types, while IFN-γ production occurs downstream through activated innate immune cells [24]. Robust IFN-β mRNA or protein expression has been reported in immune cell populations [18], ileum tissue [21], and other immune cells [22,23,24,25] within 24 h of γ-PGA exposure, though delayed IFN-β expression has been observed in PBMCs in certain scenarios [21]. In one study, IFN-β protein in lung tissue was detected only 48 h after γ-PGA administration [26]. Such variability likely reflects differences in assay methods, time points, cell types, and delivery routes. These findings underscore γ-PGA’s capacity to elicit both systemic and localized interferon responses [45,46], further supported by enhanced IFN-γ [19] and IFN-α expression [18,19,21,23]. Together, these findings highlight the broad and diverse interferon responses induced by γ-PGA.

## 6. γ-PGA and TLR4-Mediated Immune Activation: Structural Biology Perspective

TLRs are transmembrane proteins featuring an extracellular ligand recognition domain composed of leucine-rich repeats and a cytoplasmic Toll/IL-1 receptor domain [47,48]. Among them, TLR4 recognizes LPS through interactions stabilized by the co-receptor MD-2 [49,50]. Notably, only aggregated forms of LPS, rather than monomeric species, are biologically active in eliciting TLR4-mediated cytokine responses [51,52].

γ-PGA is considered a non-canonical TLR4 agonist, activating immune responses via CD14-mediated engagement of the TLR4 signaling complex [21,25]. Lee et al. [21] demonstrated that γ-PGA fails to activate TLR4 signaling in cells expressing MD-2 but lacking CD14, indicating that MD-2 alone is insufficient for γ-PGA recognition. Instead, MD-2 likely plays a supporting role in TLR4 transport to the cell surface, as TLR4 is retained in the Golgi apparatus in its absence [21].

### 6.1. Structural Docking and Recognition of γ-PGA in TLR4 Activation

**Figure 2:**
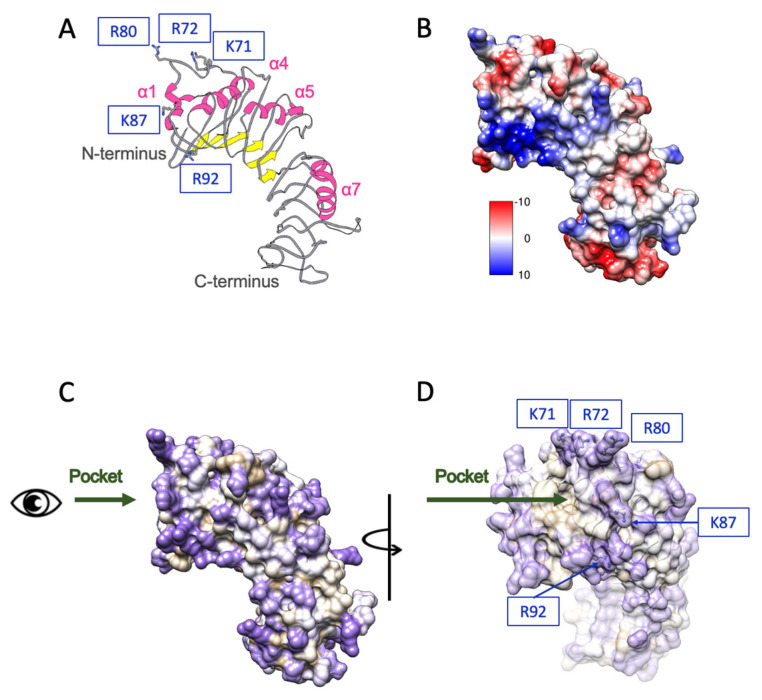
Surface and structural features of human CD14 highlighting potential γ-PGA binding sites. The crystal structure of human CD14 (PDB: 4GLP) is depicted in four panels to illustrate its structural characteristics and potential γ-PGA docking regions. (**A**) Ribbon-surface hybrid representation showing α-helices (magenta), β-strands (yellow), and overall domain architecture from the N- to C-terminus. Positively charged residues located along the rim (K71, R72) and immediately outside it (R80, K87, R92) are highlighted, suggesting potential electrostatic interactions with γ-PGA. (**B**) Electrostatic surface potential representation, with red and blue indicating negatively and positively charged areas, respectively. (**C**,**D**) Hydrophobicity surface maps presented from two different angles, with purple indicating hydrophilic and beige representing hydrophobic regions. The hydrophobic pocket is clearly visible. In (**D**), a depth-cued, semi-transparent surface overlay reveals the underlying ribbon structure, enabling clear visualization of the spatial positions of key residues. Based on spatial and electrostatic complementarity, these regions are proposed as potential docking sites for γ-PGA.

Biochemical and structural analyses have suggested that CD14 binds long, negatively charged, and hydrophilic carbohydrate chains of LPS [53,54], and CD14 also interacts with other hydrophilic, negatively charged ligands of Toll-like receptors, such as peptidoglycan and cytosine-phosphate-guanine sequences DNA, reinforcing its broad ligand specificity [55]. Structural investigations by Kelley et al. [56] identified hydrophilic binding regions on human CD14 that mediate proteoglycan recognition. These regions are supported by hydrophobicity and electrostatic surface analyses, which reveal positively charged residues—such as K71, R72, R80, K87, and R92—clustered around a hydrophilic rim near the entrance of a shallow binding pocket (Figure 2A–D). These regions are proposed to interact with diverse hydrophilic ligands such as γ-PGA. While the hydrophobic base provides structural integrity, the flexible, hydrophilic rim and adjacent grooves allow accommodation of diverse anionic polymers.

Although the exact binding mechanism of γ-PGA remains to be elucidated, its extended, negatively charged backbone is hypothesized to wrap along these charged surface patches on CD14, forming multivalent electrostatic and hydrogen-bond interactions. This surface engagement may enhance γ-PGA’s ability to promote TLR4–MD-2 clustering and downstream signaling. Unlike MD-2, which binds LPS via a deep hydrophobic pocket, CD14 provides a broad, adaptable, and electrostatically complementary interface suited for structurally flexible ligands like γ-PGA. This proposed model underscores the value of structural biology in deciphering innate immune receptor mechanisms. High-resolution cryo-electron microscopy or crystallographic studies could reveal atomic-level interactions and validate binding residues. While the extended and flexible nature of γ-PGA presents a technical challenge, this limitation may be overcome with dedicated designs tailored to stabilize its conformation during structural analysis. As an alternative, site-directed mutagenesis of surface-exposed charged grooves would be a more straightforward strategy to clarify ligand specificity and docking dynamics.

### 6.2. Structural Basis of Molecular Weight-Dependent γ-PGA Modulation of TLR4 Signaling

The immunomodulatory activity of γ-PGA is critically dependent on molecular weight, rather than its amino acid repeat sequences alone [25]. Wang et al. proposed that γ-PGA in the 30–42 kDa range adopts a spheroid-like structure composed of approximately 44% random coil, 29% β-sheet, 17% β-turns, and 10% α-helices, facilitating multivalent presentation and compact spatial organization [57]. These structural features are believed to enhance ligand–receptor engagement by increasing local concentration and spatial complementarity. Internalization of TLR4 ligands is mediated through CD14-dependent endocytosis involving both clathrin- and caveolae/lipid raft-associated pathways, which are crucial for triggering TRIF-dependent signaling [58,59,60]. γ-PGA polymers larger than 5000 kDa may form oversized aggregates that hinder cellular uptake, thereby limiting their bioavailability and signaling capacity. In contrast, low-molecular-weight γ-PGA (<500 kDa) may lack the structural integrity and surface charge density necessary for stable receptor binding or cross-linking, resulting in suboptimal immune activation [21,25]. Therefore, an optimal molecular weight window is required to achieve a balance between sufficient conformational structure and efficient internalization. This range likely enables the γ-PGA polymer to stably associate with CD14’s positively charged surface grooves (as highlighted in Figure 2) while being compact enough for endocytosis and downstream immune activation. This is supported by structural mapping of CD14, which indicates that effective ligand docking occurs at hydrophilic patches surrounded by flexible loops and positively charged residues such as K71, R72, and K87, allowing spatial accommodation of moderately sized γ-PGA polymers. Further mechanistic studies are needed to define the interplay between γ-PGA conformation, molecular weight, and receptor clustering. High-resolution docking simulations and mutagenesis targeting CD14’s binding rim will be key to understanding how different γ-PGA sizes influence receptor signaling thresholds.

## 7. Discussion

γ-PGA has emerged as a multifunctional biomaterial with strong potential for broad-spectrum antiviral applications. Its efficacy has been demonstrated across diverse in vitro and in vivo models involving clinically relevant pathogens from multiple virus families, including *Orthomyxoviridae*, *Paramyxoviridae*, *Herpesviridae*, and *Coronaviridae*. The viral entry–blocking effect of γ-PGA appears largely restricted to enveloped viruses, likely through disruption of membrane fusion, whereas its prophylactic and therapeutic actions show broader applicability, with efficacy against both enveloped and non-enveloped viruses.

Importantly, γ-PGA’s immunomodulatory activity is strongly influenced by molecular weight, which appears to affect conformation, endocytosis efficiency, and downstream type I interferon signaling [25]. This weight-dependent immune activation of γ-PGA offers a tunable parameter for optimizing antiviral efficacy. A key unresolved question is whether TLR4 activation depends solely on the intrinsic molecular weight of γ-PGA, or whether higher-order clustering (e.g., nanoparticle conjugates) is necessary to achieve the spatial threshold for CD14/TLR4 engagement.

γ-PGA induces a cascade of cytokines and establishes a robust antiviral state against diverse viruses through both TRIF-dependent and MyD88-dependent pathways. Proinflammatory cytokines, including TNF-α, IL-1α, IL-1β, IL-6, IL-12, and IFN-γ, are activated early in the immune response to recruit immune cells. Across studies, no consistent proinflammatory cytokine expression profile has been linked to γ-PGA’s molecular weight or species of subjects. Instead, cytokine kinetics and the tissue/cell-type specificity appear to be critical determinants. In particular, PBMCs and macrophage-lineage cells from humans [19] or mice [18,24] exhibit rapid upregulation of IFN-β, TNF-α, and IL-12 within hours to γ-PGA exposure. In contrast, a human epithelial cell line [18] and lung tissues from mice [26] show responses close to baseline [18,26].

Intriguingly, γ-PGA exposure seems to prime both the immune and non-immune cells and tissues. In prophylactic settings, epithelioma cells pretreated with γ-PGA produced approximately twice the amount of TNF-α during infection, compared with HSV-1 infection alone [18]. In therapeutic contexts, when γ-PGA was administered after viral exposure, a similar burst of TNF-α, along with other cytokines, was observed in pig PBMCs [23] and in murine lung tissues [26]. Notably, in the absence of viral infection, γ-PGA alone generally does not elevate systemic TNF-α levels in murine serum [21]. However, repeated injections of γ-PGA increased serum IFN-β levels by day 7 in pigs, as reported by Seo et al. [23]. Overall, in most animal models, prophylactic γ-PGA treatment dose not lead to massive systemic cytokine production unless viral exposure occurs.

Despite encouraging immunological data, the molecular basis for γ-PGA’s interaction with innate immune receptors remains structurally undefined. Structural biology holds the key to unraveling these mechanisms. Cryo-electron microscopy or X-ray crystallographic studies of the γ-PGA/CD14/TLR4–MD-2 complex are essential to reveal binding interactions at atomic resolution. Given MD-2’s limited binding to γ-PGA [53], it is hypothesized that γ-PGA’s polyanionic chains dock on positively charged lysine- and arginine-rich surfaces of CD14, promoting multivalent clustering and spatial proximity to the TLR4–MD-2 heterodimer. However, this remains a conceptual model, as no experimentally resolved structure of the ternary or quaternary γ-PGA/CD14/TLR4 complex is currently available. Mapping these interactions structurally would help clarify how γ-PGA initiates downstream signaling.

Moreover, biophysical studies such as isothermal titration calorimetry, surface plasmon resonance, or cryo-EM of reconstituted complexes could be employed to determine binding stoichiometry and affinity. Future mutagenesis studies targeting CD14’s polysaccharide-binding domains could validate proposed binding sites and define the structural features necessary for immunostimulatory activity. Such investigations would further delineate the thresholds of ligand size, charge density, and multivalent binding required for optimal receptor activation. These limitations also highlight an urgent need to define whether TLR4 activation by γ-PGA is direct, cooperative, or secondary to conformational changes in CD14. Identifying these determinants will enable a more mechanistic understanding of immune potentiation.

Addressing these structural questions will be critical for rational design of γ-PGA-based antivirals with enhanced potency, selectivity, and delivery profiles. Owing to its biodegradability, safety profile, and versatility, γ-PGA is a promising platform for next-generation antiviral strategies. Neither the 5-day repeated dosing study in mice nor the 4–8-week clinical trials in humans demonstrated evidence of TLR4 tolerance, chronic inflammation, or immune exhaustion, and short- to mid-term safety was favorable [21,31,32]. Nevertheless, uncertainty remains owing to the lack of data on treatment durations beyond three months. Overall, its prophylactic efficacy against diverse viruses supports its potential as a preventive agent, consistent with the concept of innate immune priming or “trained immunity”.

## 8. Conclusions

The reproducibility of these results across models and laboratories underscores the potential of γ-PGA as a broad-spectrum antiviral, acting through three main mechanisms: (1) blocking viral entry in enveloped viruses, (2) prophylactic immune priming via TLR4/CD14 signaling, including IFN-β and ISGs, and (3) therapeutic immune activation post-infection, characterized by a surge of IFN-β expression. Its efficacy is strongly influenced by molecular weight (~890–3000 kDa). Mechanistic insights from structural biology were also highlighted. γ-PGA may interact with CD14–TLR4–MD-2 complex through electrostatic binding to CD14 surface grooves, driving immune activation.

Despite these encouraging findings, several critical gaps remain: (1) Mechanistic understanding of γ-PGA’s receptor interactions, particularly its docking with CD14 and TLR4, requires continued structural, biochemical, and mutational analyses. (2) Expanding preclinical testing to additional viral models, including non-enveloped viruses and emerging pathogens, will further clarify its breadth of action. (3) Systematic studies are needed to evaluate its long-term safety, cytokine kinetics, and risk of hyperinflammation. (4) Direct evidence of the antiviral effect of γ-PGA on clinical trials is also required to confirm efficacy, durability of protection, and translational applicability.

Overall, γ-PGA combines structural tunability, broad-spectrum antiviral activity, and a strong safety record, positioning it as a next-generation antiviral and immunomodulatory platform. Continued structural, biochemical, and clinical research will be key to translating this potential into therapeutic reality.

## Figures and Tables

**Table 1:** Reported in vitro and in vivo antiviral activities of γ-PGA against various viruses in three mechanisms.

Mechanisms	Authors	Target Virus	M.W., Dose of γ-PGA, and Formulations	Routes and Models	Cytokines and Signaling Pathways
Interference in viral entry	Sánchez-León et al. [17]	HSV-1, HSV-2, VSV, PRV	5 kDa, 5 μg/mL Extracellular polymer comprising γ-PGA and teichoic acid	in vitro: γ-PGA was mixed with virus stock for 1 h and then added to cells for another hour. After two washes to remove unbound virus, viral titers were assessed 24 h post the viral inoculation (Vero, HOG, Mewo, Hela, Jurkat).	It exhibits a dose-dependent virucidal effect via blocking viral entry.
Interference in viral entry; therapeutic treatment	Poli et al. [19]	HSV-2	890–1000 kDa, 0.4 mg/mL Purified γ-PGA	in vitro: PBMCs were co-treated with γ-PGA and HSV-2 without washing.	It upregulates cytokines including IFN-γ, IFN-α, TNF-α, IL-12, and IL-18.
Prophylactic treatment	Moon et al. [22]	Influenza A (H1N1 and H5N2)	>3000 kDa, 1 mg/mL Commercially purified	in vitro: RAW 264.7 cells were pretreated with γ-PGA for 12 h prior to the infection. in vivo: Mice received intranasal γ-PGA 12 h prior to viral challenge.	It upregulates IFN-β, IFN-α, and TNF-α mRNA in lung tissue and increases ISG mRNA expression in macrophages.
Prophylactic treatment	Talactac et al. [24]	NDV	>3000 kDa, 1 mg/mL Commercially purified	in vitro: RAW 264.7 cells were pretreated with γ-PGA 12 h prior to viral challenge.	It upregulates IRF3, IFN-β, Mx1, and other ISGs and inhibits virus budding. Cytokine profile resembles LPS-treated cells. Inhibits virus budding.
Prophylactic treatment	Marino-Merlo et al. [18]	HSV-1	890–1000 kDa, 0.5 mg/mL Purified γ-PGA	in vitro: Hep-2 cells were treated with γ-PGA 2 h before viral challenge.	It upregulates cytokines including NF-κB, TNF-α, and IL-1β.
Prophylactic treatment	Lee et al. [21]	MNV	2000 kDa, 100 nM, 50 mg/kg of body weight Commercially purified	in vitro: RAW 264.7 cells pretreated with γ-PGA 6 h before the infection. in vivo: Mice received daily oral γ-PGA for 5 days before viral challenge.	It blocks viral entry, induces IFN-γ, and elevates IFN-β in serum and ileum without triggering proinflammatory cytokines.
Prophylactic and therapeutic treatment	Lee et al. [25]	SARS-CoV, HCV	2000 kDa, 100 μM Commercially purified	in vitro: Hek293-TLR4 cells were treated with γ-PGA 12 h after transfection with the SARS-CoV replicon. in vitro: Hu7 cells were transfected with the HCV genome for 24 h, followed by γ-PGA treatment for 18 h.	γ-PGA acts as a TLR4 ligand, requiring both CD14 and MD-2 to activate IFN-β mRNA expression.
Therapeutic treatment	Kim et al. [26]	Influenza A H1N1	2000 kDa, 100 μg in 20 μL PBS Commercially purified	in vivo: Mice received intranasal γ-PGA 24 h after viral inoculation.	It enhances antiviral cytokines (IFN-β, IL-12) and activates NK cells as well as influenza-specific cytotoxic T cells.
Therapeutic treatment	Seo et al. [23]	PRRSV	>2000 kDa, 20 mg/mL Commercially purified	in vivo: Pigs received intramuscular γ-PGA 7 days after viral challenge.	It upregulates cytokines including TNF-α, IFN-α, IFN-β, IFN-γ, IL-2, IL-4, and IL-6.

Note: γ-PGA, gamma-poly(glutamic acid); CD14, cluster of differentiation 14; HCV, hepatitis C virus; HSV, herpes simplex virus; IFN-α, interferon-alpha; IFN-β, interferon-beta; IFN-γ, interferon-gamma; IL, interleukin; IL-1β, interleukin-1 beta; IL-2, interleukin-2; IL-4, interleukin-4; IL-6, interleukin-6; IL-12, interleukin-12; IL-18, interleukin-18; IRF3, interferon regulatory factor 3; ISG, interferon-stimulated gene; LPS, lipopolysaccharide; Mx1, myxovirus resistance protein 1; NDV, Newcastle disease virus; NF-κB, nuclear factor kappa-light-chain-enhancer of activated B cells; NK, natural killer; PBS, phosphate-buffered saline; PRRSV, porcine reproductive and respiratory syndrome virus; PRV, pseudorabies virus; SARS-CoV, severe acute respiratory syndrome coronavirus; TLR4, toll-like receptor 4; TNF-α, tumor necrosis factor-alpha; VSV, vesicular stomatitis virus. Cell lines: Hek293, human embryonic kidney 293 cells; Hela, human cervical cancer cells; Hep-2, human epithelial type 2 cells; HOG, human oligodendroglioma cells; Hu7, human hepatoma cells; Jurkat, human T lymphocyte cells; Mewo, human melanoma cells; PBMCs, peripheral blood mononuclear cells; RAW 264.7, murine macrophages; Vero, African green monkey kidney cells.

## Data Availability

No new data were created or analyzed in this study. Data sharing is not applicable to this article.

## Abbreviations

The following abbreviations are used in this manuscript:

γ-PGA	γ-poly(glutamic acid)
CD14	Cluster of differentiation 14
H1N1	Influenza A virus subtype H1N1
H5N2	Influenza A virus subtype H5N2
HCV	Hepatitis C virus
Hek293	Human embryonic kidney 293 cells
Hela	Human cervical cancer cells
Hep-2	Human epithelial type 2 cells
HOG	Human oligodendroglioma cells
HPV	Human papillomavirus
HSV-1	Herpes simplex virus type 1
HSV-2	Herpes simplex virus type 2
Hu7	Human hepatoma cells
IFN-α	Interferon-alpha
IFN-β	Interferon-beta
IFN-γ	Interferon-gamma
IKKβ	Iκb kinase β
IL-1β	Interleukin-1 beta
IL-2	Interleukin-2
IL-4	Interleukin-4
IL-6	Interleukin-6
IL-12	Interleukin-12
IL-18	Interleukin-18
IRAKs	Interleukin-1 receptor–associated kinases
IRF3	Interferon regulatory factor 3
IRF7	Interferon regulatory factor 7
ISG(s)	Interferon-Stimulated Gene(s)
ISRE	Interferon-stimulated response element
Jurkat	Human T lymphocyte cells
kDa	Kilodalton
LPS	Lipopolysaccharide
MD-2	Myeloid differentiation factor 2
Mewo	Human melanoma cells
MNV	Murine norovirus
Mx1	Myxovirus resistance protein 1
MyD88	Myeloid differentiation primary response 88
NDV	Newcastle disease virus
NF-κB	Nuclear factor kappa-light-chain-enhancer of activated b cells
NK cells	Natural killer cells
OAS	2′-5′-oligoadenylate synthetase
PBMCs	Peripheral blood mononuclear cells
PBS	Phosphate-buffered saline
PRRSV	Porcine reproductive and respiratory syndrome virus
PRV	Pseudorabies virus
RAW 264.7	Murine macrophage cells
SARS-CoV	Severe acute respiratory syndrome coronavirus
TLR4	Toll-like receptor 4
TNF-α	Tumor necrosis factor-alpha
TRAF6	Tumor necrosis factor receptor-associated factor 6
TRAFs	Tumor necrosis factor receptor-associated factors
TRAM	TRIF-related adaptor molecule
TRIF	TIR-domain–containing adaptor inducing IFN-β
Vero	African green monkey kidney cells
VSV	Vesicular stomatitis virus
WISH	Human amnion cells
